# Radical or Not-So-Radical Prostatectomy: Do Surgical Margins Matter?

**DOI:** 10.3390/cancers14010013

**Published:** 2021-12-21

**Authors:** Ioanna Maria Grypari, Vasiliki Zolota, Vasiliki Tzelepi

**Affiliations:** Department of Pathology, School of Medicine, University of Patras, 26504 Patras, Greece; med5812@upnet.gr (I.M.G.); zol@med.upatras.gr (V.Z.)

**Keywords:** prostate cancer, margins of resection, radical prostatectomy, prognosis, pathology

## Abstract

**Simple Summary:**

Prostate cancer is the second most common noncutaneous malignancy in men. Prostatectomy is a commonly used treatment modality for selected patients. The prostate’s ill-defined borders and its vicinity with vital structures complicate the wide excision of the organ, resulting in positive margins of resection. Neoplastic infiltration of margins of resection in prostatectomy specimens affects patients’ prognosis. The surgical technique and surgeons’ expertise affect the incidence of margin positivity. The location and the extent of positive margins diversify the risk of recurrence, with basal infiltration and multifocal foci of positive margins behaving more aggressively. Pathologists are encouraged to thoroughly report the status of margins of resection, as they provide important information for patients’ prognosis and enable the clinician to decide upon the most appropriate subsequent therapeutic steps.

**Abstract:**

Prostate cancer is the second most common malignancy in men, and prostatectomy is the treatment of choice for most patients with at least low risk of progression. The presence of positive margins in the radical prostatectomy specimen is considered an adverse pathologic feature, and may prompt additional therapeutic intervention in the patients. The absence of a distinct capsule around the prostate and intraoperative manipulations that aim to minimize postoperative adverse effects, complicate its wide removal. Proper handling of the specimen during the gross processing is essential for accurate determination of the status of margins or resection. Positive margins, defined as the presence of neoplastic glands in the highlighted-with-ink margin of resection, range from 6–38%. The surgical technique, surgeon’s expertise and tumor (i.e., grade and stage) and patients’ (i.e., BMI) characteristics affect the rate of margin positivity. Extensive or multifocal and nonanterior/nonapical positive margins are linked with higher recurrence rates, especially in organ-confined disease, underscoring the need for treating these patients more aggressively. In summary, detailed description of the status of the margins should be performed in every pathology report to determine patients’ prognosis and the most appropriate therapeutic plan.

## 1. Introduction

Prostate cancer is the second most common noncutaneous malignancy in men (the first most common in half of the countries of the world, including those in Europe and North America) with 1,414,259 new cases worldwide in 2020, and represents the fifth most common cause of cancer death in men [1,2]. There is a great diversity in the clinical outcome of patients with PCa. Some patients have indolent cancer that will never progress, whereas others have remarkably aggressive disease with rapid progression to metastases and resistance to therapy [3,4]. In between are patients with initially localized disease that will progress to metastatic incurable disease after a variable time period [3].

Currently, prostate-specific antigen (PSA) levels, clinical stage and biopsy Prognostic Grade Group [5] are the parameters used to stratify patients into risk categories (very low, low, intermediate, high and very high), albeit with modest specificity and sensitivity, and to decide on the appropriate therapy [6,7]. The intermediate risk group is further subdivided into favorable and unfavorable subgroups based on the number of intermediate risk features present, the grade group and the number of positive cores [7].

According to the NCCN guidelines [7], radical prostatectomy may be offered to patients with very low-risk disease and life expectancy >20 years and those with low risk disease and life expectancy of >10 years, although active surveillance is the preferred treatment option in both groups of patients. Radical prostatectomy may be offered in patients under active surveillance upon disease progression. Radical prostatectomy may also be performed in patients with favorable intermediate-risk disease and life expectancy >10 years (along with pelvic lymph node dissection—PLND—if predicted probability of lymph node metastasis is ≥2%). Active surveillance and EBRT or brachytherapy are alternative options in this risk group. Radical prostatectomy is a therapeutic option for patients with unfavorable intermediate risk group and life expectancy >10 years (along with PLND if predicted probability of lymph node metastasis is ≥2%) and for patients with high or very high risk and life expectancy >5 years or symptomatic (along with PLND). EBRT plus androgen deprivation therapy (ADT) or EBRT + brachytherapy +/− ADT are alternative options for these patients (+/− docetaxel for very high-risk patients). Salvage radical prostatectomy may also be performed for patients with biochemical recurrence after initial EBRT, but morbidity is significant in this setting [8,9].

The presence of positive margins in the radical prostatectomy specimen is considered an adverse pathologic feature, and may prompt additional therapeutic intervention in the patients, like EBRT +/− ADT [7]. In an attempt to elucidate the significance of surgical margins in prostatectomy specimens, a review of the literature was performed, focusing on the association of positive surgical margins with adverse pathology parameters and patients’ prognosis.

## 2. Pathology

The prostate is partially enveloped by a capsule, consisting of a layer of smooth muscle bundles mainly arranged in a transverse plane, and collagen fibers, usually concentrated as a thin line at the external border [10,11]. The smooth muscle cells of the internal border of the capsule blend with the prostatic stroma [11]. The thickness of the capsule, the relative proportions of collagen and smooth muscle and the distance of the prostatic acini to the surface of the prostate vary significantly, even within different areas of the same gland [10]. Additionally, the capsule is missing in the anterior and anterolateral surfaces of the gland, even though its outer collagen region is consistently identified in most parts of the gland. [11] Thus, the prostatic capsule cannot be regarded as a well-defined anatomic structure, and the terms extracapsular extension, invasion of the capsule and capsule penetration are no longer accepted in prostatectomy reports, being replaced by the term extraprostatic extension [12,13].

**Figure 1:**
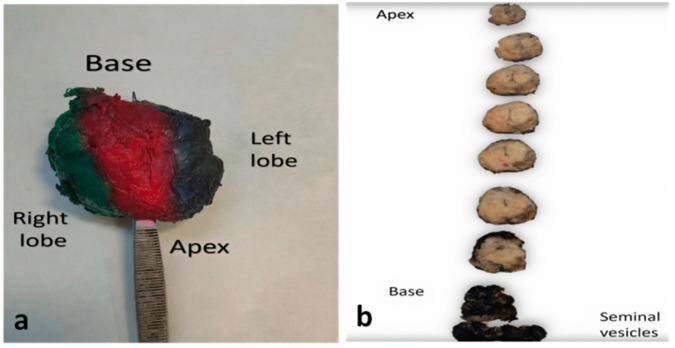
Gross handling of prostatectomy specimens (**a**) Prostate specimen, colored with different colors in each surface to help orientation. (**b**) Serial sectioning through the transverse axis from apex to base (margins colored with blank ink). Apex and base will be subsequently sectioned in a parallel (parasagittal) way (not shown).

To ensure precise assessment of the margins, at the Pathology Department, the whole surface of the prostate is inked with one or multiple colors depending on the pathologist preference and the department’s protocol (Figure 1a) [14]. Special considerations should be given to the grossing of the apex and the base. To reduce overestimation of positive margins, the cone method or a parallel/parasagittal sectioning is preferred for both anatomical areas over the trimming of a save block (Figure 1b) [14].

**Figure 2:**
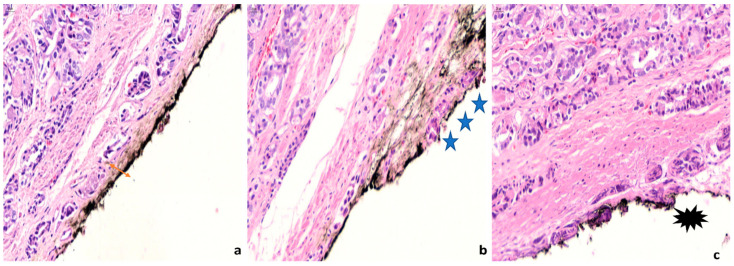
Definition of margin positivity (**a**) Neoplastic cells are close but not in contact with the ink. The distance between the inked margin and the neoplastic cells is pointed with a double-sided arrow. (**b**) Neoplastic cells are in contact with the ink. This is considered a positive margin. Three asterisks indicate the area of margin infiltration. (**c**) Cautery artifact in neoplastic cells in contact with the ink. An asterisk highlights the area of cautery effect. (400× magnification).

A positive margin of resection (MOR) is defined as malignant glands in contact with the ink (Figure 2) [14], which means that the neoplasm extends to the area where the surgeon has dissected the tissue [15]. The presence of neoplastic cells close but not in contact with the surface of the prostate is not considered as a positive margin (Figure 2) no matter how close the cells are to the ink [16], as studies have shown that this has no prognostic significance [17,18].

Practical issues regarding what is considered a positive margin are common. In the anterior surface of the prostate, the gland is surrounded by a fibromuscular stroma and its capsule is incomplete, thus, there is increased difficulty in recognizing the presence of extraprostatic extension and positive MOR [19]. In addition, cautery artifacts are frequent at the MORs (Figure 2) and may rarely pose difficulty in determining the nature of the cells that are present at the ink. 

## 3. Factors Affecting the Probability of Margin Positivity

The frequency of positive MOR in prostatectomy specimens ranges from 6–38%, depending on a variety of factors, including the stage of the disease, the surgical technique used and the experience of the surgeon [20,21]. Interestingly, the frequency of margin positivity has declined during the period 1982–2001 from 40% to almost 10%. This may be attributed to the fact that tumors are being diagnosed at an earlier stage compared to the past. In addition, discrepancies in the recognition and report system of surgical margins in the pathology report may also account for the differences across the years [21].

A positive margin may be created in two ways: failure of the surgeon to fully excise the extraprostatic extension of the neoplasm in a tumor that is locally advanced (at least pT3a) and intraprostatic incision of a T2 tumor. Vital structures are present in the immediate vicinity of the gland and the periprostatic tissue is rather scant. In addition, the prostate capsule is not a well-defined anatomic structure, and a definite plane of section is not present. Thus, the dissection may be deliberately performed as close to the prostate surface as possible to avoid damaging the adjacent vital structures or the lack of plane of section may result in intraprostatic excision [16]. Τhe surgical technique used correlates with the probability of a positive margin. A challenge for the urologist is to minimize positive MOR, while retaining the maximal length of urethra and the neurovascular bundles, reducing the adverse effects of prostatectomy such as incontinence and impotence [15].

The incidence of positive MOR ranges from 6–38% in robotic and open prostatectomies [22,23,24]. A lower risk of positive margins has been shown for robotic assisted prostatectomy compared to the open surgical technique in a prospective study [25], especially in low stage disease and in experienced surgeons [26]. A systematic meta-analysis of 19 observational studies, however, showed that open and robotic technique have no difference in terms of risk of margin positivity [27]. Similarly, a randomized controlled phase 3 study failed to show any difference between the two techniques [28], although, this study has been criticized for design flaws [29].

The wide surgical resection of prostate entails cutting posterior to the Denonvillier’s fascia and into the perirectal fat, along the neurovascular bundles [22]. In the nerve-sparing technique, dissection is performed very close to the prostate surface and the neurovascular bundles are spared to minimize the risk for erectile dysfunction and urinary incontinence [30,31], factors affecting quality of life after surgery. It is not surprising that the wide resection technique is linked with significantly lower rates of positive MOR compared with the nerve-sparing technique [32] lowering the risk of positive MOR by 60%, regardless of the tumor characteristics [22].

Retzius-sparing robotic-assisted prostatectomy, also called posterior approach, is a surgical technique where the excision of the prostate is performed through dissection of the posterior space of Douglas, leaving the retropubic space of Retzius intact [33]. This technique has better outcomes for localized tumors in regard to urinary continence shortly after the surgery [33], compared to the anterior approach, as the puboprostatic ligaments and endopelvic fascia are preserved [33]. However, a higher rate of positive MOR with Retzius-sparing robotic-assisted prostatectomy compared to the traditional technique has been shown in some studies [33], albeit the level of evidence is quite low and other studies have shown no difference between the two techniques [34].

The patient’s characteristics may also influence the frequency of positive margins. Increased BMI is considered a surgical challenge, however, it is linked with a lower risk of positive MOR because the prostate in this setting is surrounded by plenty of fat, facilitating the surgical excision of the gland with a safe margin [23,24,35]. Additionally, total testosterone levels have been associated with positive MOR rates [35,36]. In contrast, age >70 years old has not been associated with probability of positive margins in a retrospective cohort of patients treated with robotic radical prostatectomy [37].

The level of surgeon’s expertise has a crucial role in resecting the entire prostate with negative margins [23,24,33,38]. It has been calculated that performance of 200–250 cases is required in order for the surgeon to acquire the essential experience to perform this type of surgery [21,36]. Experienced surgeons have lower rates of positive margins, leading to reduced biochemical recurrence rates [23]. Surgeons with limited experience, that is, performance of 50–60 surgeries, have increased rates of positive MOR, even in organ-confined disease [35].

Lastly, and as expected, the frequency of margin positivity is affected by the tumor characteristics. The possibility of positive MOR ranges from 5–30% for pT2 (organ-confined) disease, to 17–65% for pT3 neoplasms [39,40,41,42].

Independent factors associated with positive MOR are body mass index (BMI), PSA and high D’Amico score [36]. Factors that predict non focal positive MOR are the number of positive cores in preoperative biopsy, stage pT3a and pT3b [24]. In contrast, Zhang et al. conducted a comprehensive meta-analysis and systematic review with high diversity and a sample of 50,014 patients, and showed that age, BMI, prostate volume and type of surgical technique are not reliable and independent prognostic factors for margin positivity [43]. They confirmed, however, that preoperative PSA levels, Gleason score at the initial diagnostic biopsy, Gleason score and stage at the prostatectomy specimen, and presence of positive lymph nodes, extraprostatic extension and seminal vesicle invasion are associated with positive MOR [43].

## 4. Clinical Significance of Positive Margins

Presence of positive margins is an independent adverse prognostic factor for recurrence [36,40,41,42,44], especially when accompanied by simultaneous EPE [19] and has been associated with higher mortality [45,46], although it does not seem to be the most important factor predicting cancer-related death [46]. Biochemical recurrence-free probability of patients with pT2 disease and positive margins has been shown to be similar to those of patients with T3a disease and negative margins. The same applies to patients with T3a and positive margins and those with T3b and negative margins [44]. Surprisingly, in pT3b stage, margin status was not predictive of biochemical recurrence [41], probably because the disease is driven by the aggressive nature of the neoplastic cells and not the positive margins. In contrast, in organ-confined disease, positive margins are associated with reduced biochemical recurrence-free survival independently of other adverse pathology characteristics [47]. Although data from prospective studies are lacking, these findings have been verified in a meta-analysis that included 41 retrospective cohort studies [48].

It is widely accepted that margin positivity is an important feature for deciding upon the next step after surgery [46,49]. Results from multi-institutional randomized trials have shown that there is a benefit of radiotherapy compared with active surveillance after prostatectomy regarding biochemical recurrence-free survival in patients with positive margins, though the overall survival is not improved [41]. In addition, margin status seems to be the best predictor of the positive effect of adjuvant radiotherapy after prostatectomy [50]. According to the latest guidelines of the European Society of Urologists, patients that show two of the three high-grade features (positive margins, pT3 stage and grade group 4 or 5) are candidates for adjuvant radiation therapy [20]. Similarly, the NCCN guidelines consider positive margins after radical prostatectomy, along with seminal vesicle invasion, extracapsular extension and detectable PSA as an indication for adjuvant radiotherapy [7].

## 5. Pathologic Features of Positive Margins with Clinical Significance

The significance of marginal invasion is not always predictive of patients’ clinical outcome, as prognosis depends on a variety of factors. This means that some positive margins will be a source of recurrence for the patient, whereas in other cases the neoplastic cells at the margin will not have any clinical significance. This is further supported by the fact that patients with positive margins after surgery that demonstrate undetectable PSA levels at its nadir have better biochemical recurrence-free survival compared to those with a detectable PSA nadir, and patients with positive margins that their PSA reaches its nadir at 3 months have better biochemical recurrence-free survival than patients that reached the PSA nadir in more than 6 months [49]. Studies have shown that it is not only the presence, but also the extent and location of the positive margins, as well as the Gleason Score of the tumor at the positive margin that is of significance, and may guide therapeutic interventions [51]. The latest College of American Pathologists protocols (revised in June 2021) have embodied the above-mentioned features in the pathology report [12].

**Figure 3:**
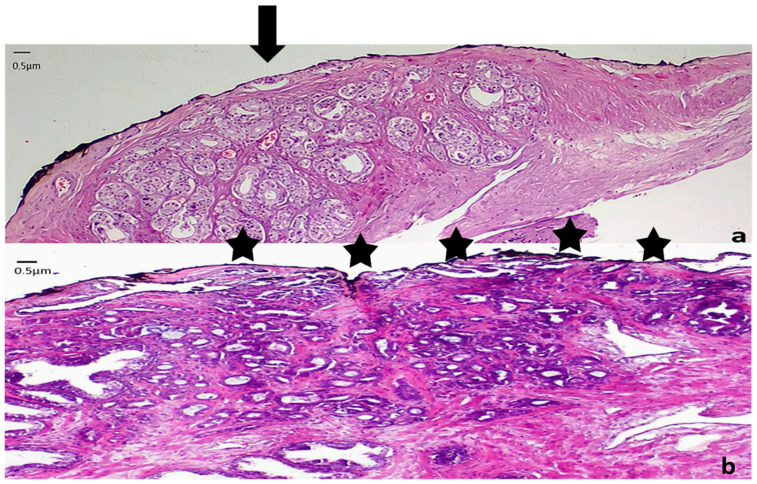
Representative images from low grade carcinomas with positive margins of different extent (**a**) Focal margin infiltration with neoplasm graded as PGG1. An arrow indicates the area of margin positivity (100× magnification) (**b**) Nonfocal margin infiltration with neoplasm graded as PGG1. Five asterisks point the area of margin positivity. (40× magnification).

The length and number of areas with positive margins of resection have been correlated with the rate of biochemical recurrence [39,41,46,51,52,53,54,55,56]. A cutoff of 3 mm of linear extent of positive margins has been used in many studies to distinguish limited from extensive margin positivity, and is the cutoff recommended by the College of American Pathologists protocol [12]. Koskas et al. in a retroperspective study with an 8-year period of follow up demonstrated that extended or multifocal positive MOR is linked with earlier and higher rates of recurrence compared to limited positive margins [40]. They also found that Gleason score or pT stage does not influence in a statistical significant way the length of positive margins, whereas focal marginal infiltration does not affect the rate of biochemical recurrence [40]. Similarly, it has been shown that focal infiltration of MOR correlates with biochemical but not with clinical recurrence [20]. Extensive invasion of surgical margins is accompanied by a 35% chance of 5-year recurrence-free survival, compared with 60% for limited MOR and 87% for prostatectomies with organ-confined disease [16]. More recently, a lack of a significant difference in biochemical recurrence between patients with negative and short (<3 mm) (Figure 3) positive MOR has been shown [24,57]. In addition, when invasion of margins is unifocal, the biochemical-free period is not shortened [24,40]. The limited prognostic value of short and unifocal positive MOR may be accounted by a potential false interpretation of the margin, due to artifacts during the handling of the specimens. In addition, the few neoplastic cells remaining in the margins may not be not able to multiply and metastasize [57], and the cautery and ischemic effect of surgery may have destroyed the limited amount of neoplasm that has remained in the patient [16]. Taking into account these findings, the extension and the topography of positive MOR should be seriously considered when planning therapeutic interventions, as it is considered an independent adverse prognostic factor [44]. NCCN define as diffuse margins that are >10 mm or involving ≥ 3 sites [7]. A consideration for adjuvant treatment should be given for patients with extensive positive margins [20].

Gleason score of the tumor at the positive margin has also been associated with the prognosis [58,59,60,61], albeit in some studies it has been found that it is not an independent factor [47]. A recent meta-analysis of 10 retrospective cohort studies, found that primary Gleason grade 4 or 5 at the margin was predictive of biochemical-free recurrence [62]. Prognostic grade group (PGG) 1 at the margin has been shown to have biochemical recurrence rates [60] and cancer-specific survival [61] equivalent to those of negative margins. Paradoxically, when Gleason score 6 tumors are accompanied by extensive (>3 mm) positive MOR, there might be an increased risk of recurrence [52]. That means that PGG1 tumor at the margin, especially when limited in extent, is not capable of progression, in concordance with the indolent nature of Gleason 6 tumors [63]. In contrast, PGG2-5 tumors at the margin have a higher rate of biochemical recurrence compared to PGG1 [62], and the presence of Gleason grade 4 at the margin in patients with Gleason score 7 tumors is associated with worse cancer-specific survival compared to the presence of Gleason grade 3 at the margin, independently of the tumor’s Gleason score and the use of adjuvant therapy [61]. These data support the report of the Gleason Score of the neoplastic glands that are present at the margin in the pathology assessment of prostatectomy specimens, also recommended by the College of American Pathologists [12].

Probability of margin positivity differs in the different areas of the prostate, as does its prognostic significance. Apex is the most common location for positive MOR, followed by the posterolateral and the posterior surface [16,19,21,36,40,41,52]. The frequent positivity of the margins at the apex is attributed to the lack of a clear plane of resection in this area, its close proximity to vital structures such as the dorsal venous complex and the neurovascular bundles and the agony of the surgeon to preserve the maximum length of the urethra, thus limiting the urological complications postoperatively [21]. In addition, the apex is surrounded by a very thin and fragile capsule where the benign glands are admixed with skeletal muscles, thus, it is prone to fragmentation during its handling by the surgeon [21]. The lack of periprostatic adipose tissue and the interminglement and graduate transition of the prostate parenchyma to the external urethral sphincter at the apex [10] make the definition of margin positivity and whether it is related to extraprostatic extension or intraprostatic excision difficult in this area [16].

Interestingly, the apical–anterior positive margins have lower prognostic significance than the nonapical/nonanterior [32] or basal [19,56] margins, even though this has been disputed [55]. Similarly, it has been noticed that positive margins in the region of bladder neck or base of the prostate are associated with concurrent positive margins in other regions [15]. The more indolent behavior of positive anterior/apical margins may be explained by the different grade and molecular background of tumors arising in the different areas of the prostate. PTEN deletions, a genetic abnormality commonly seen in later stages of PCa progression [64], are less frequent in PCa arising in the transition zone compared to peripheral zone tumors [65] and anterior tumors are of lower grade compared to posterior zone tumors [66]. This underscores the complexity of prostate cancer biology and the heterogeneity of patients’ clinical outcome. Further studies should be performed to strengthen the association of the extent, as well as the topography of PSM separately in each pathology stage to precisely select patients with maximum benefit from adjuvant therapy.

## 6. Conclusions

In conclusion, the presence of positive margins is an important pathologic parameter, and along with other features determines patient’s prognosis and the need for adjuvant therapy. The clinical significance of margin positivity depends on various factors, including its location and extent, tumor stage and grade, and the grade of the tumor at the margin. The clinical significance of positive margins is higher when they are extensive or multifocal, and when the tumor is high-stage and high-grade. However, and as mentioned above, positive margins may predict biochemical recurrence even in patients with organ-confined disease and Gleason score ≤7. Thus, detailed description of margin status should be performed in every pathology report to help clinicians decide upon the most appropriate next therapeutic steps.
